# Multi-Modal Assessment of Long-Term Erythropoietin Treatment after Neonatal Hypoxic-Ischemic Injury in Rat Brain

**DOI:** 10.1371/journal.pone.0095643

**Published:** 2014-04-22

**Authors:** Yohan van de Looij, Alexandra Chatagner, Charles Quairiaux, Rolf Gruetter, Petra S. Hüppi, Stéphane V. Sizonenko

**Affiliations:** 1 Division of Child Development and Growth, Department of Paediatrics, School of Medicine, University of Geneva and Geneva University Hospital, Geneva, Switzerland; 2 Laboratory for Functional and Metabolic Imaging (LIFMET), Ecole Polytechnique Fédérale de Lausanne (EPFL), Lausanne, Switzerland; 3 Department of Fundamental Neurosciences, School of Medicine, University of Geneva, Geneva, Switzerland; 4 Department of Radiology, University of Lausanne, Lausanne, Switzerland; 5 Department of Radiology, University of Geneva, Geneva, Switzerland; Robert Debre Hospital, France

## Abstract

Erythropoietin (EPO) has been recognized as a neuroprotective agent. In animal models of neonatal brain injury, exogenous EPO has been shown to reduce lesion size, improve structure and function. Experimental studies have focused on short course treatment after injury. Timing, dose and length of treatment in preterm brain damage remain to be defined. We have evaluated the effects of high dose and long-term EPO treatment in hypoxic-ischemic (HI) injury in 3 days old (P3) rat pups using histopathology, magnetic resonance imaging (MRI) and spectroscopy (MRS) as well as functional assessment with somatosensory-evoked potentials (SEP). After HI, rat pups were assessed by MRI for initial damage and were randomized to receive EPO or vehicle. At the end of treatment period (P25) the size of resulting cortical damage and white matter (WM) microstructure integrity were assessed by MRI and cortical metabolism by MRS. Whisker elicited SEP were recorded to evaluate somatosensory function. Brains were collected for neuropathological assessment. The EPO treated animals did not show significant decrease of the HI induced cortical loss at P25. WM microstructure measured by diffusion tensor imaging was improved and SEP response in the injured cortex was recovered in the EPO treated animals compared to vehicle treated animals. In addition, the metabolic profile was less altered in the EPO group. Long-term treatment with high dose EPO after HI injury in the very immature rat brain induced recovery of WM microstructure and connectivity as well as somatosensory cortical function despite no effects on volume of cortical damage. This indicates that long-term high-dose EPO induces recovery of structural and functional connectivity despite persisting gross anatomical cortical alteration resulting from HI.

## Introduction

Neonatal hypoxic-ischemic (HI) brain injuries involve both primary destructive events including neuronal death, gliotic reaction and also secondary maturational disturbances leading to subsequent abnormal development of cerebral white and gray matter [1], [2]. These damages lead to altered function that range from severe neurological handicap to behavioral, learning and attention deficits [2]. The postnatal day 3 (P3) rat shares some similarities in terms of cortical neuronal, glial and oligodendroglial development to the very preterm infant (24–28 weeks) [3]. P3-HI injury damages specific brain regions and is characterized by diffuse intracortical white matter loss, zones of patchy neuronal degeneration, altered oligodendrocyte progenitors maturation and subsequent myelination, and hypertrophic astrocytes with formation of gliotic scares [4]–[7]. These neuropathological features are mainly found in the somatosensory cortex. In direct correlation with the neonatal HI damage of the deep infragranular cortical area, including the subplate neurons area, P3-HI injured rats show profound functional alteration of neuronal response with a reduced somatosensory amplitude response to whisker stimulus contralateral to the lesioned hemisphere. However, in the first 3 weeks after birth, partial recovery processes take place in the establishment of the sensorimotor cortical networks during the cortical functional maturation period [8].

Erythropoietin (EPO) is a 30.5-kDa cytokine functioning mainly as a survival and differentiation regulator during erythropoiesis [9]. In the central nervous system, neurons, glial cells and cerebral endothelial cells produce EPO and express EPO-receptors (EPO-R) [9]. EPO has been shown to act as an anti-apoptotic, anti-inflammatory and neurotrophic mediator both *in-vitro* and *in-vivo* [9], [10]. Several studies documented an autocrine production of EPO not only by astrocytes but also by neurons during conditions of suffering of the cerebral tissue [11], [12]. Hypoxic stress increases the production of EPO by the neuronal cells in culture [13]. Further, EPO treatment of neurons in culture reverted glutamate-induced cell death [14]. In p7 rats, infarct size as a result from neonatal stroke induced by middle cerebral artery occlusion was reduced by EPO administration [15]. In neonatal mice and rats EPO pre-treatment or early after HI insult was also neuroprotective when assessed early at 2–7 days after insult [16]–[18]. Finally, the neuroprotective effect of EPO treatment after neonatal HI was reported to include a preservation of tyrosine hydroxylase-positive fibers in the ventral tegmental area and substantia nigra pars compacta [19]. EPO has also shown anti-apoptotic effects on oligodendrocytes and promoted neurogenesis and angiogenesis, which are essential for normal neurodevelopment but also injury repair [20], [21]. Reducing the initial injury and supporting the subsequent development through neurotrophic factors appear both important mechanisms in neonatal brain injury [22], [23].

Magnetic Resonance Imaging (MRI) and Spectroscopy (MRS) are non-invasive MR techniques widely used to assess brain structure and brain metabolism, respectively. Anatomic T_2_-Weighted MR images lead to accurate quantification of brain region volumes and has been used to assess lesion volume early after HI as well as long term cortical loss [24]. Diffusion tensor imaging (DTI) is a powerful tool to probe cerebral white matter through a 3D description of water diffusion in the tissue [25]. Changes in diffusivity values (mean: MD, parallel: D_//_ and orthogonal: D_⊥_) and/or fractional anisotropy (FA) derived from DTI reveal loss of brain microstructure integrity [26], [27]. ^1^H magnetic resonance spectroscopy (^1^H-MRS) has been used to follow the changes in “neurochemical profile” of the brain during cerebral development [28]. This technique allows assessment of several metabolite concentrations (named as the “neurochemical profile”) known to be altered following HI in the P3 pup rat [24]. Recently, a minimally invasive epicranial approach for multielectrode recordings of vibrissal somatosensory-evoked potentials (SEPs) applied to the P3 neonatal HI model revealed long-term functional deficit [8] and EPO treatment showed sensorimotor recovery after HI injury in the neonatal mice and in spinal cord injury [29], [30].

EPO has been shown efficient in different models of term and moderately preterm brain injury [31], [32] but has not been used in a very immature P3 HI model. Further, only short-term protective treatment regimens have been evaluated. In the present study, we investigated the neuroprotective effects, including damage reduction and neurotrophic action, of long-term high dose EPO administration in the P3-HI model using a multi-modal strategy that combine histological, macro-structural (MRI), micro-structural (DTI), neurochemical (MRS) as well as functional (SEP) assessments.

## Materials and Methods

All animal experiments were approved by the Swiss Veterinary Office and in adequacy with Swiss animal welfare laws. The unilateral P3 moderate HI model has been described previously [6], [7]. Briefly, P3 male Wistar pups underwent right carotid artery cauterization under isoflurane anesthesia then after 30 minutes recovery rat pups were exposed to 6% O_2_ hypoxia for 30 minutes under thermoneutral conditions.

### Magnetic Resonance Imaging: T_2_-Weighted Images

All *in-vivo* MR experiments were performed on an actively-shielded 9.4T/31 cm magnet (Magnex Scientific, Abington, UK; Varian, Palo Alto, CA, USA) equipped with 12-cm gradient coils (400 mT/m, 120 µs) with a quadrature transceive 17-mm surface RF coil. During measurements, each pup was placed supine within an adapted holder and continuously anesthetized under a flow of 1.5–2% isoflurane in oxygen. Body temperature was maintained at 37°C using thermoregulated water circulation. 5 h following HI, T_2_W Fast Spin Echo (FSE) images with TE/TR = 80/6000 ms; FOV = 25×25 mm and matrix size = 256×128 were used to detect presence of injury as well as determinate the initial lesion volume. Only the pups showing hyper-intense signal in the ipsilateral hemisphere on these acute T_2_W images were randomized for the therapeutic intervention.

### EPO Treatment

Animals showing injury on initial MRI were selected and randomized to NaCl group, injected intraperitoneally with NaCl 0.9% or to EPO group, injected intraperitoneally with recombinant human EPO (rhEPO, Roche, Basel, Switzerland) 10 U/g body weight/day during the first week after HI (P3 to P10) and then 5 U/g body weight 3×/week until P25. A Control group, which was not subjected to surgery and injection, was also studied.

### ^1^H Magnetic Resonance Spectroscopy

To study the effects of the treatment on brain metabolic profile, 22 days following HI (P25), ^1^H-MRS was performed. At P25, FSE images using the same parameters as at P3 were performed for positioning of ^1^H-MRS voxel of interest (1.5×1.5×2.5 mm^3^). Spectra acquisition was performed as previously described [24] using an ultra-short echo time (TE/TR = 2.7/4000 ms) SPECIAL spectroscopy method [33]. Measurements were made in the area of somatosensory cortex as reported previously [5]–[7], [24] as well as the corresponding cortical area for the Control group (n = 8 rats for each group, NaCl, EPO and Control). 70 series of FIDs (12 averages each) were acquired, individually corrected for frequency drift, summed together and corrected for residual eddy current effects using the reference water signal. Proton spectra were analyzed with LCModel [34] as previously described [24]. Metabolites were quantified resulting in a neurochemical profile of the cortical lesion for the EPO and NaCl groups and of the cortex in the Control group.

### Volumetric Measurements

From FSE images, volumetric measurements were performed using Anatomist/Brain Visa [35]. At P3, lesion volume (LV) as well as ipsilateral cortical volume (ICV) was measured; at P25 contralateral cortical volume (CCV) as well as ipsilateral cortical volume (ICV) was quantified. Volumetric measurements resulted in percentage of injured cortex ( = (LV/ICV)×100) at P3 and percentage of cortical loss ( = [(CCV-ICV)/CCV]×100) at P25 (n = 8 rats for each group, NaCl and EPO).

### Somatosensory-evoked Potentials (SEPs) Recordings

Following ^1^H*-*MRS, epicranial recordings of somatosensory evoked potentials (SEP) were carried out at P27–P28 under isoflurane anesthesia as previously described [8] (n = 11, 10 and 9 rats for NaCl, EPO and Control group, respectively). Before surgery, anesthesia was induced with 3–4% isoflurane then rats were mounted in a stereotaxic frame. Isoflurane was lowered at 2.5% and local bipuvacaine anesthesia was applied to the scalp. The skull was exposed by retracting the skin from the frontal to the occipital bones. A 16 steel electrodes grid dipped in EEG paste (500 µm in diameter, final impedance ≈ 50 kOhms) was applied on the skull, covering the entire exposed surface (see [8] for electrode coordinates). SEP were recorded at 0.7–1% isoflurane with a custom-made amplifier (gain 5000X; band pass filters 1–500 Hz, analog to digital conversion at 2 KHz [8]). During recordings, absence of withdrawal hind limb reflex was controlled and body temperature was maintained at 37°C using a heating pad.

Unilateral somatosensory stimuli were delivered simultaneously to all large whiskers on one side of the snout through a solenoid-based stimulator device [8], [36]. Stimuli consisted of 500 µm back-and-forth deflections with 1 ms rise time. Right-sided and left-sided series of 50 stimuli were applied with an inter-stimulus interval of 9 seconds.

Individual SEPs were calculated offline by averaging responses 100 ms prestimulus to 500 ms poststimulus. Maximum positive voltage peaks were measured for each SEP at right and left S1 electrodes (e4, e12).

### Ex-vivo Diffusion Tensor Imaging

Following SEPs, animals received an overdose of intraperitoneal injection of sodium pentobarbital and were transcardially perfused with NaCl 0.9% followed by with 4% paraformaldehyde on 0.1% PBS for tissue fixation. DTI experiments on *ex-vivo* brain were performed with a transceiver 25-mm birdcage RF coil (n = 6 rats for each group, NaCl, EPO and Control). Spin Echo sequence (TE/TR = 30/5000 ms) was used, diffusion gradients were applied along dual gradient diffusion gradient sampling scheme [25] (G_diff_ = 22 G/cm, δ = 3 ms and Δ = 20 ms given a *b*-value of 1184 s.mm^−2^). 20 slices of 0.8 mm thickness with an in-plane pixel size of 70 µm were acquired in the axial plane. Using a homemade Matlab (Mathworks, Natick, MA, USA) software [27], [37], region of interest (ROI) were manually delineated on four different regions of the brain: the external capsule (EC), the internal capsule (IC), the corpus callosum (CC) and the superficial layer of sensorimotor cortex (Cx) at four different levels of the brain corresponding to the genu, the body (2 different parts of the body) and the splenium of the corpus callosum.

### Immunohistology

Ex-vivo imaged brains added with non-scanned ones at P25 were used for immunohistological studies (n = 8 rats for each group, NaCl, EPO and Control). Anti-Glial fibrillary acid protein (anti-GFAP, Dako, ZO334, Glastrip, Denmark) and anti-Neuronal Nuclei (anti-NeuN, Chemicon, Billerica, MA, USA) staining were used to observe the long-term effects of EPO on astrocytes activation, resulting in formation of gliotic scare and on neuronal death and survival, respectively. Anti-Myelin basic protein antibody (anti-MBP, Chemicon, Billerica, MA, USA) was used to determine white matter injury with altered myelination. Anti-Neurofilament antibody (anti-SMI 31, Chemicon, Billerica, MA, USA) was used to assess neurofilament integrity.

After brain fixation as previously described, pup brains were cryoprotected in 30% sucrose and frozen. Contiguous 12 µm sections at the level of the dorsal hippocampus were cut on a cryostat and collected on superfrost slides (ThermoScientific, Waltham, MA, USA). Immunostaining was performed using the following protocol: sections were washed in a 0.1% PBS and non-specific binding was blocked by incubating the slides in 0.1% PBS/5% BSA/0.3% triton/3% normal goat serum (NGS). They were then incubated overnight at 4°C with the different primary antibodies: NeuN 1∶200 in 0.1 M PBS/pH 7.4/5% BSA/1% NGS, GFAP 1∶400 in 0.1 M PBS/pH 7.4/5% BSA/1% NGS, MBP 1∶200 in 0.1 M PBS/pH 7.4/5% BSA/1% NGS and SMI 31 1∶200 in 0.1 M PBS/pH 7.4/5% BSA/1% NGS. This was followed by 2 hours incubation at room temperature with secondary goat Alexa 488 anti-rabbit 1/1000 (Lifetechnologies, Carlsbad, CA, USA) for GFAP and Goat Alexa 555 anti-Mouse 1/1000 (Lifetechnologies, Carlsbad, CA, USA) for NeuN, MBP and SMI 31 in 0.1 M PBS/pH 7.4/5% BSA/1% NGS. Slides were finally washed and cover slipped with mounting media.

For each specific staining and each brain, three slides of 220 µm sections apart were scanned using the Mirax (Zeiss, Göttingen, Germany) and analyzed. GFAP-positive staining was evaluated in the ipsilateral cortex of HI animals. A scoring system with scores ranging from 0 (no gliotic scare) to 4 (strong staining covering the whole surface of parietal cortex in the deeper layers) was used.

Other immunostaining were evaluated in Metamorph (Molecular Devices, Sunnyvale, CA, USA) using specific automated journals defined for desired unbiased quantification. The quantification of neurons was done by counting DAPI and NeuN positive cells in both cortices of each animal for the three groups leading to number of neurons in the ipsilateral and contralateral cortices for each group (NnI and NnC, respectively). The percentage of neuronal loss in the ipsilateral cortex was then calculated using the formula: ((NnC–NnI)/(NnC+NnI))×100, for each group.

The same automated journal was used to quantify length of myelinated fibers (from MBP staining) and neurite outgrowth (from SMI 31 staining). Briefly, first the skeletons of the fibers were detected and measured and, second the distance from the cortex surface to the extremity of the fibers was measured. Finally, the second distance was subtracted to the first one, resulting in a fiber length taking into account the cortical thickness. Resulted lengths were all summed together leading to total length of myelinated fibers and total length of neurofilaments in the ipsilateral cortex for each rat of the three groups.

The average fluorescence intensity for MBP and SMI 31 positive staining deducted from the background was assessed in the entire surface of ipsilateral cortices within each animal of the three groups. In the genu of the corpus callosum, in the internal and external capsule, three ROI having the same surface were drawn in each zone and immunofluorescence was measured. The background was subtracted and the mean of the three zones was calculated, indicating the level of myelination and of intact neurofilaments in these areas.

### Statistical Analysis

All the data are presented as mean ± standard deviation (SD) except for SEP results presented as mean ± standard error of the mean (SEM). For MRI-MRS and immunohistology, a Wilcoxon test was used to compare values between ipsilateral and contralateral sides of the same animal and a Mann-Whitney test to compare statistically values between the groups (n = 8 for each group), both using Matlab (Mathworks, Natik, MA, USA). For SEPs, as voltage peaks and ratio distributions turned out to be normal, ANOVA and t-tests were applied for between and within group comparisons. Analyses were performed using Cartool (http://brainmapping.unige.ch/Cartool.htm) and Matlab (Mathworks, Natik, MA, USA).

## Results

At the end of the treatment period, hemoglobin and hematocite were measured in both NaCl and EPO treated animals and no difference was seen.

### Astroglial Scar Formation

GFAP-positive staining was present in the ipsilateral cortex of HI animals. Various degrees of astrogliosis in layers IV, V, VI in the parietal cortex of the right injured hemisphere were observed. In the NaCl group, 28.55% of animals showed a score of 0, 14.25% a score of 1, 28.55% a score of 2 and 28.55% a score of 3. In the EPO group, 42.85% showed a score of 0, 14.25% a score of 1, 28.55% a score of 3 and 14.25% a score of 4. No significant score difference was found between NaCl and EPO groups.

### Neuronal Loss Assessment

Quantification of neurons revealed a significant increase of neuronal loss in NaCl compared to Control groups (19.1±13.9% *vs.* 0.9±1.4%, respectively; *P*≤0.0001) as well as between EPO and Control groups (30.9% ±25.1 *vs.* 0.9% ±1.4 *P*≤0.0001). No significant difference was observed between EPO and NaCl groups.

### Myelin Staining

**Figure 1:**
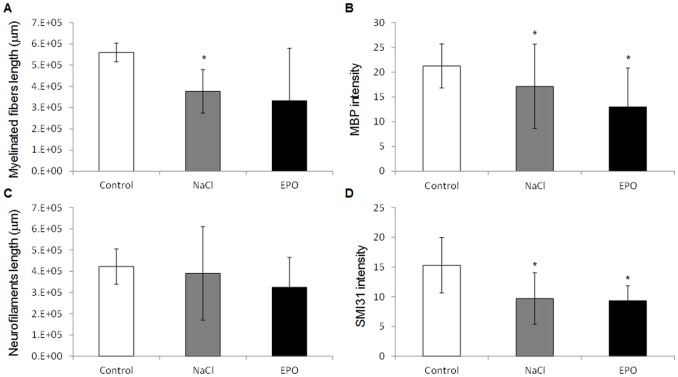
Myelination and neurofilaments in the ipsilateral cortex. A) Total length of myelinated fibers (µm) of MBP positive stained fibers in the cortex of Control, NaCl and EPO treated rats. B) MBP average fluorescence intensity in the cortex of Control, NaCl and EPO treated rats. C) Total length of neurofilaments (µm) of SMI31 stained fibers in the cortex of Control, NaCl and EPO treated rats. D) SMI31 average fluorescence intensity in the cortex of Control, NaCl and EPO treated rats. All the data are presented as mean ± SD, *: *P*<0.05, NaCl *vs.* Control and EPO *vs.* Control, n = 12 rats for each group, NaCl, EPO and Control.

In the cortex, myelinisation along the fibers measured as the total cortical MBP positive fibers (figure 1A) was reduced in NaCl compared to Control groups (376768 µm ±102627 and 559701 µm ±43771, respectively; *P* = 0.007) while there was no significant difference neither between EPO and Control groups (331953±247332 µm and 559701±43771 µm, respectively) nor between NaCl and EPO groups.

The average fluorescent intensity of MBP staining (figure 1B) was significantly reduced in the EPO compared to Control (12.98±7.85 and 21.25±4.43, respectively; *P* = 0.014) as well as in the NaCl compared to Control groups (17.13±8.5 and 21.25±4.43, respectively; *P* = 0.011). EPO group *vs.* NaCl group presented no significant difference. In the corpus callosum, in the external and internal capsules, the quantifications of the MBP fluorescent signal did not reveal any significant difference between the groups Control, NaCl and EPO (data not shown).

### Neurofilaments

In the cortex, the analysis of neurofilaments length (figure 1C) did not reveal any significant difference between NaCl, EPO and Control groups (324970±140475 µm, 390235±220066 µm and 422241±82999 µm, respectively). In the other hand, the average fluorescent intensity of SMI 31 staining (figure 1D) was significantly reduced in the EPO compared to Control groups (9.32±2.51 *vs*. 15.29±4.66, respectively; *P* = 0.04) as well as between the NaCl and Control groups (9.69±6.38 *vs*. 15.29±4.66, respectively; *P* = 0.02). There was no significant difference between NaCl and EPO groups. No significant difference was observed in the corpus callosum, external and internal capsules, in SMI 31 quantifications between the three groups (data not shown).

### Cortical Volumes

**Figure 2:**
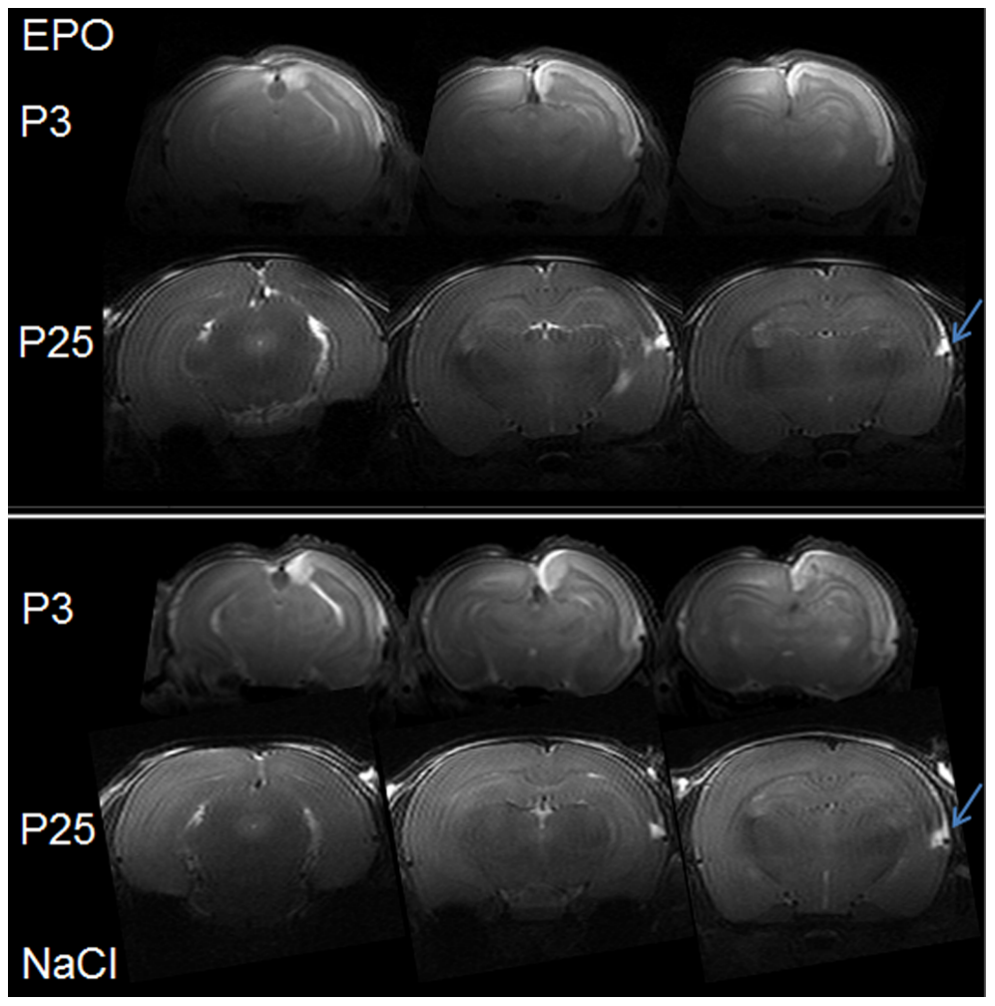
T_2_ weighted MRI at P3 and P25. T_2_ weighted images of typical EPO and NaCl treated pup rats at 5 h (P3) and 22 d (P25) after injury showing the evolution of the ischemic lesion in the ipsilateral cortex (right). At P3, ischemic lesion appears as hypersignal. At P25, images present a large cortical loss in the ipsilateral cortex and an abnormal development for the both groups. Remaining trace of lesion was observed for the both groups at the bottom of the cortex (arrows).

**Figure 3:**
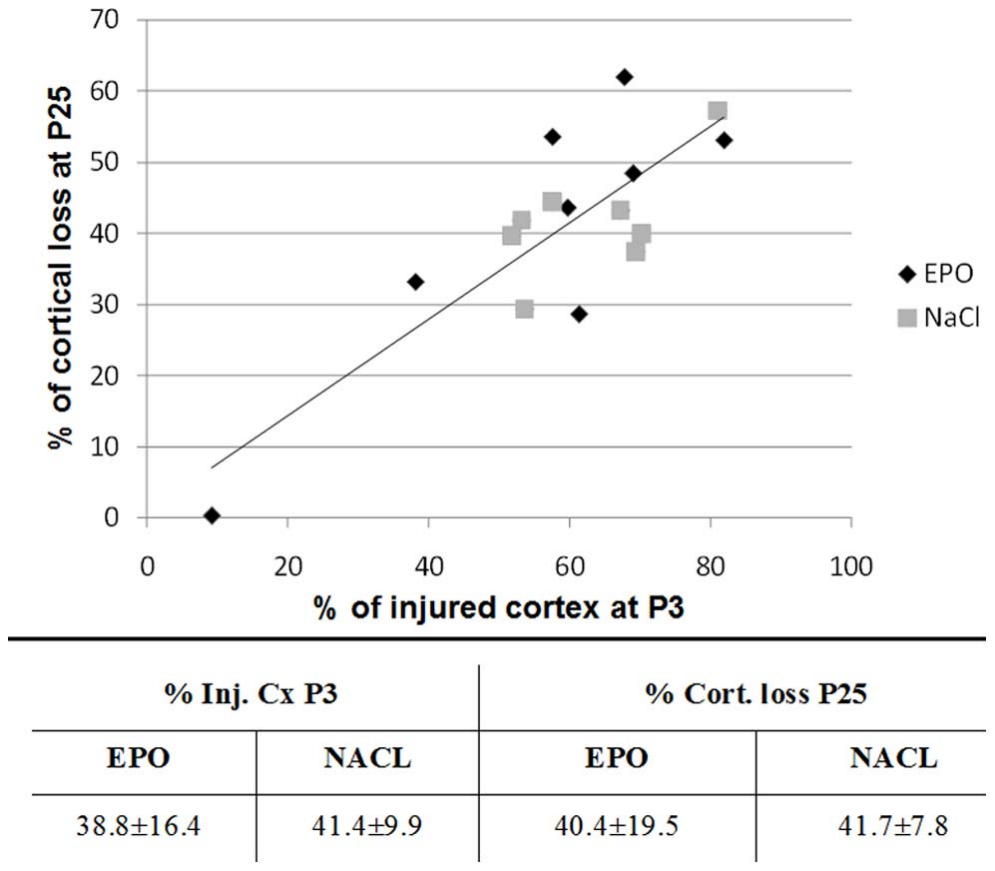
Volumes of cortical injury at P3 and loss at P25. Upper panel: percentage of cortical loss at P25 as a function of the percentage of injured cortex at P3 for the both groups: EPO and NaCl. A strong correlation independent of the group was found: R^2^ = 0.67; *P* = 0.0001. Lower panel: percentage of injured cortex (Inj. Cx) at P3 and percentage of cortical loss (% Cort. loss) at P25 for EPO and NaCl groups. There were no statistical differences between EPO and NaCl groups in terms of cortical injury at P3 and loss at P25. Data are presented as mean ± SD, n = 8 rats for each group, NaCl, EPO and Control.

Figure 2 shows multi-slice T_2_-weighted FSE brain images of a typical EPO and NaCl treated pup rat at different time points (5 h (P3) and 22 d (P25) after injury) showing the evolution of the ischemic lesion in the ipsilateral cortex for the both groups. At P3, ischemic lesion appears in hypersignal due to the presence of edema (figure 2). At P25 for the both groups, a small residual T_2_ hypersignal was observed (figure 2, arrows) at the level of the rhinal fissure. No cyst was present but images showed cortical loss in the ipsilateral hemisphere in both groups. There was no difference in the percentage of injured cortex at P3 before treatment randomization between EPO and NaCl groups (38.8±16.4% *vs.* 41.4±9.9% for EPO and NaCl, respectively) showing the homogeneity of injury in both groups. A strong correlation independent of the groups was found between the percentage of injured cortex at P3 and the percentage of cortical loss at P25 (figure 3, R^2^ = 0.67; *P* = 0.0001). At P25, the percentage of cortical loss was not different between EPO and NaCl treated pups (40.4±19.5% *vs.* 41.7±7.8% for EPO and NaCl, respectively).

### Metabolic Profile of Cortex

**Figure 4:**
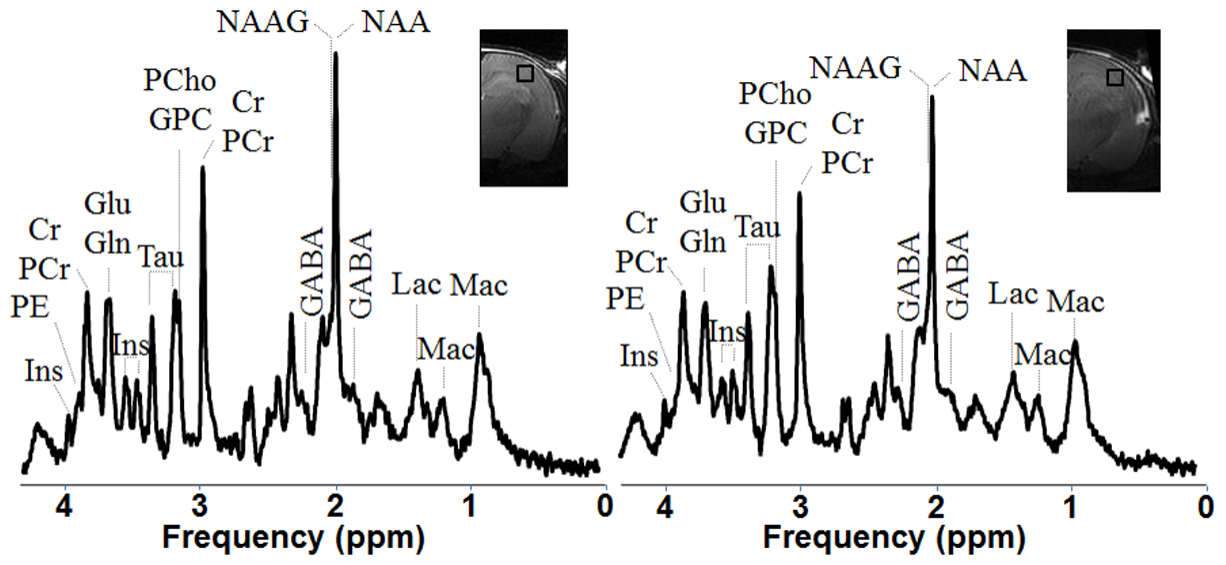
Metabolic profile of cortex. Typical in^1^H-NMR spectra at 9.4T in the ipsilateral cortex of one NaCl treated rat pup (right) as well as in the same area of a Control rat cortex with respective metabolite assignments. All spectra (SPECIAL, TE/TR = 2.8/4000 ms, NT = 840) were displayed with Gaussian apodization (gf = 0.11 s) and scaled relative to the concentration of macromolecules. Abbreviations: Mac, macromolecules; Asc, ascorbate; Asp**,** aspartate; GPC, Glycero-phosphocholine; PCho, phosphorylcholine; Cr, creatine; PCr, phosphocreatine; GABA, γ-aminobuttyric acid; Glc, glucose; Glu, glutamate; Gln, glutamine, Ins, myo-inositol; Lac, lactate; NAA, N-acetylaspartate; NAAG, N-acetylaspartylglutamate; PE, phosphoethanolamine and Tau, taurine.

Good spectral quality was achieved in the current study, as judged from water linewidth, obtained with FASTMAP, ranging from 8 Hz to 12 Hz. Due to very thin cortical structure in the rat pup brain, MRS was performed on a very small volume of 12 µl placed on the cortex (figure 4). On the overall study signal to noise ratio was equal in average to 15±3. These consistent data were subjected to spectral analysis and absolute quantification using an LCModel. It should be noticed that the voxel of interest was always placed higher than the remaining T_2_ hypersignal previously described, avoiding a possible water contamination in the metabolite quantifications.

Table 1 summarizes the changes observed on metabolite concentrations. Comparing ipsilateral and control cortices, for both the NaCl group and the EPO group significant decrease of [Glu] as well as [totalNAA], [Glu]+[Gln] and [totalCr] was observed in the lesion area. In addition in NaCl group, [NAA], [Cr] and [PCr] were also significantly decreased in the injured cortex. Ipsilateral cortical “neurochemical profiles” of EPO versus NaCl did not show any significant difference.

**Table 1:** Neurochemical profiles.

	EPO	vs. CTL	NACL	vs. CTL	CTL
**Mac**	1.8±0.1		1.8±0.1		1.9±0.2
**Cr**	3.1±0.5		3.0±0.5	*↓	3.6±0.5
**PCr**	4.5±2.2		3.5±0.6	**↓	4.2±0.5
**Glc**	4.2±3.2		2.9±0.8		3.7±3.1
**Gln**	2.6±0.6		2.9±0.4	*↓	3.3±1.0
**Glu**	7.1±0.9	**↓	6.9±1.6	*↓	9.3±1.3
**NAA**	7.6±1.4		7.2±0.9	**↓	9.0±0.8
**Tau**	6.5±1.2		6.2±1.7		7.5±0.6
**NAA+NAAG**	8.3±1.3	*↓	8.0±0.9	**↓	9.9±1.0
**Glu+Gln**	9.6±1.0	***↓	9.8±1.8	*↓	12.6±1.8
**GPC+PCho**	0.5±0.2		0.6±0.2		0.6±0.2
**Cr+PCr**	7.3±1.7	*↓	6.5±0.9	**↓	7.9±0.8
**PCr/Cr**	1.1±0.2		1.2±0.3		1.2±0.2
**Glu/Gln**	2.9±0.9		2.4±0.5		3.0±0.9

Metabolite concentrations in the ipsilateral cortex of EPO and NaCl treated rat pups as well as in the control cortex, and summary of observed metabolite changes following HI in the EPO and NaCl treated rats when compared to the control rats. Changes are displayed with specific orientated arrows: “↑”, increase and “↓”, decrease. The number of “*” represents significant level of *P*<0.05, 0.01, 0.001 respectively. Notice that no significant difference was found between EPO and NaCl cortices. All concentrations are presented as mean ± SD in mM/g except the ratios. Abbreviations: Mac, macromolecules; Asc, ascorbate; Asp**,** aspartate; GPC, Glycero-phosphocholine; PCho, phosphorylcholine; Cr, creatine; PCr, phosphocreatine; GABA, γ-aminobuttyric acid; Glc, glucose; Glu, glutamate; Gln, glutamine, Ins, myo-inositol; Lac, lactate; NAA, N-acetylaspartate; NAAG, N-acetylaspartylglutamate; PE, phosphoethanolamine and Tau, taurine.

### Somatosensory Evoked Potentials

**Figure 5:**
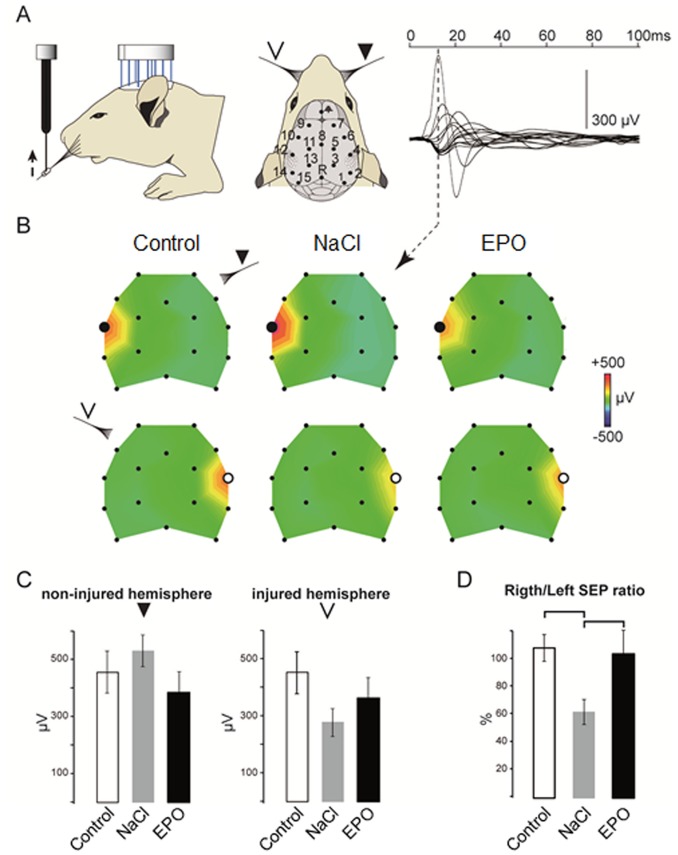
Functional evaluation with SEP. A. Design of the epicranial recording setup. SEPs were recorded in response to unilateral left-sided or right-sided whisker stimuli using an array of 16 epicranial electrodes (n = 11, 10 and 9 rats for NaCl, EPO and Control group, respectively). On the right, an example of the average superimposed SEP traces of all electrodes recorded in one control animal in response to 50 right whisker stimuli. The dashed line indicates the time of the peak positive response typically recorded at the contralateral S1 electrode and characterizing the first SEP component. B. Color-coded voltage maps at the time of the peak response of the grand average for right-sided (upper row) and left-sided (lower row) stimulation in the Control, NaCl and EPO groups. C. Histograms showing for all groups the mean ± SEM voltage amplitude for the peak SEP response at the S1 electrode (white dots) in the non-injured (right, e12) and injured (left, e4) hemispheres in response to contralateral stimuli. D. Mean ± SEM ratios calculated between peak SEP amplitudes at the S1 electrodes.

To analyze the effect of the EPO treatment on functional recovery, we recorded SEP in response to whisker stimulation (figure 5A). As already described in previous studies [8], the earliest and strongest SEP component in response to unilateral whisker stimulation shows the activation of the contralateral S1 region in control and in HI groups (figure 5B). The topographic voltage maps at the peak amplitude of this SEP component suggest a deficit in the amplitude of the response to left-sided whisker stimuli in the right S1 cortices of the NaCl as compared to the control and EPO groups as well as to the response to right-sided whisker stimuli in their left, non-injured, hemisphere. No significant difference was found in the mean voltage peak amplitudes recorded above S1 in the non-injured hemisphere between the 3 groups (Mean ± SEM: control: 461±84 µV; NaCl = 507±94 µV; EPO = 388±83 µV; *P* = 0.65, one-way ANOVA; figure 5C). However the SEP peak amplitude is greatly reduced in the right injured hemisphere of the NaCl group compared to the control group with the EPO group showing an intermediate SEP peak amplitude reduction (Mean ± SEM: control: 463±73 µV; NaCl = 291±58 µV; EPO = 359±53 µV). ANOVA analyses failed to show a group effect because of the variability of the SEP voltage values between individuals. However, within group comparisons using paired statistics revealed in the animals of the NaCl group a strong and significant reduction of contralateral SEP amplitude in the right hemisphere as compared to contralateral SEP in their left hemisphere (*P* = 0.02, paired t-test, two tailed). No difference was found between contralateral SEPs both in the control and in the EPO groups. Accordingly, the mean SEP ratios between right and left peak amplitudes was significantly different between groups (*P* = 0.04; ANOVA): while the right/left ratio is around 100% in the control and the EPO groups, a strong bias is observed in the NaCl group, with a mean value in the injured hemisphere reaching only 62±9% of the contralateral side (Figure 5D). Such a bias indicates reduced amplitude of the response in the injured hemisphere however a mechanism of compensation in the contralateral non-injured hemisphere may also participate to the bias of the SEP in the NaCl group following the unilateral lesion.

### Microstructure Evaluation by DTI

**Figure 6:**
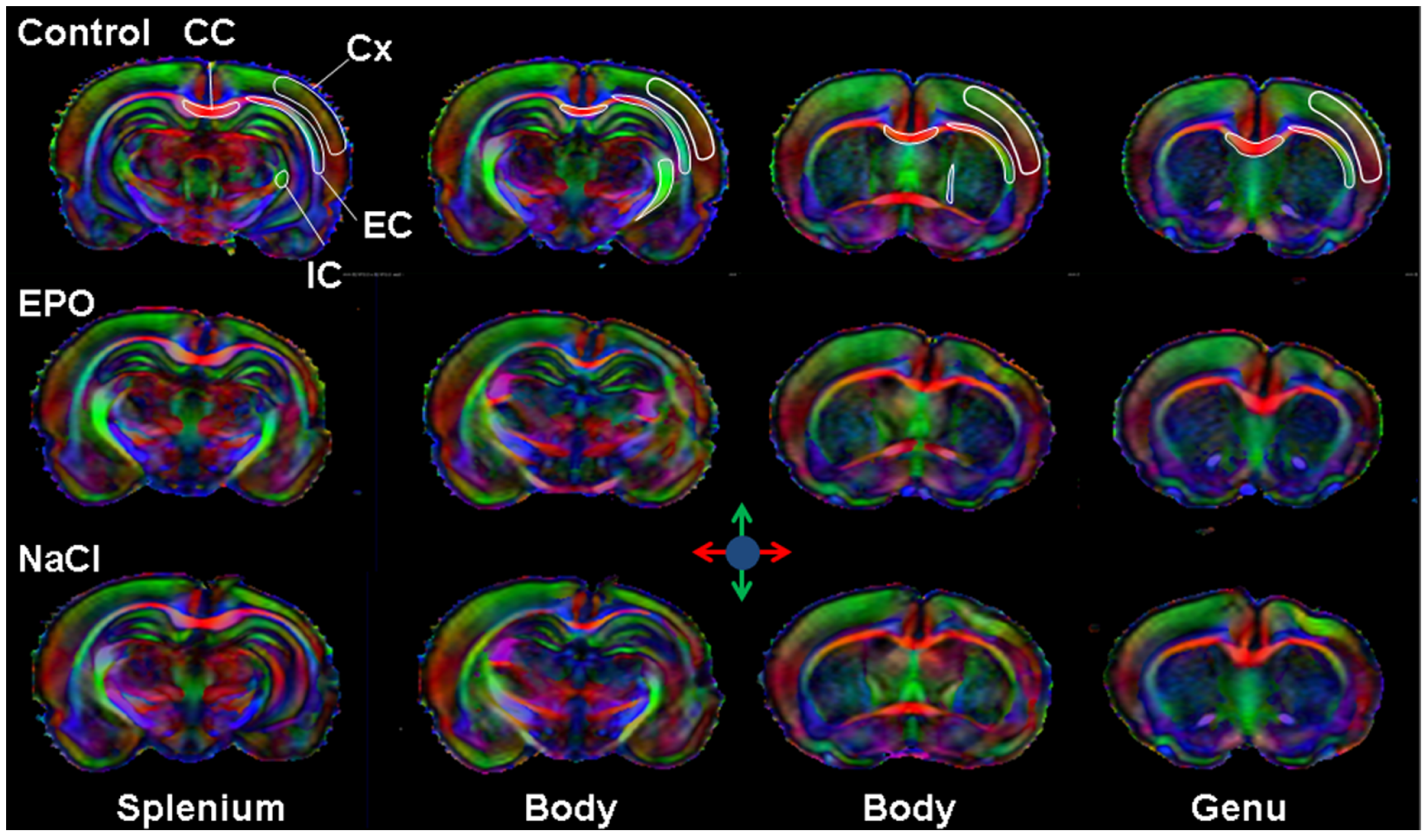
DTI analysis of microstructure. Direction encoded color maps of typical P25 Control, EPO and NaCl pup rat brain at four different image-planes corresponding to the four levels of DTI indices measurements: Splenium, Body, Body and Genu of the corpus callosum. The different ROIs analyzed are overlaid on the Control map: external capsule (EC), internal capsule (IC), corpus callosum (CC) and cortex (Cx).

Direction encoded brain color maps of a typical EPO and NaCl treated pup rats are presented on figure 6. The excellent SNR and resolution (70 µm in-plane) of these images allowed an accurate estimation of diffusion tensor derived parameters. No obvious visual differences appeared between the two maps. Abnormal development was obvious for both rats with a dissymmetry between ipsilateral and contralateral hemispheres (*i.e.* thinner cortex in the injured hemisphere due to cortical loss following injury). The contralateral hemisphere was comparable to the one of the control group (not shown). It should be noticed that these diffusion data were acquired on *ex-vivo* fixed brain, thus the diffusivity values are significantly lower than *in-vivo* acquisition whereas FA values remained unchanged after fixation as previously described [38].

**Figure 7:**
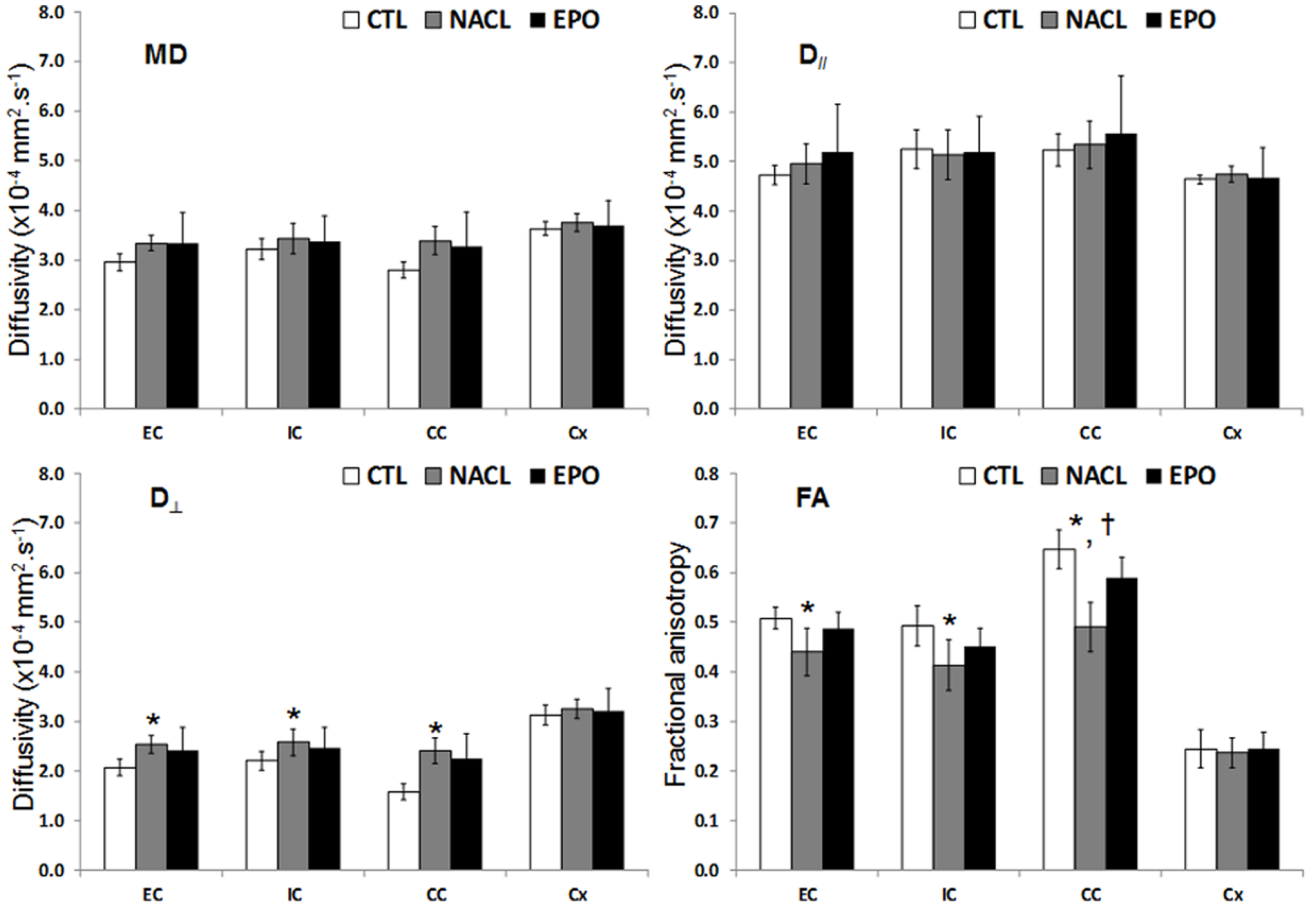
Microstructure assessment with DTI. Histogram of diffusivities (Mean: MD, axial: D_//_and radial: D_⊥_) as well as fractional anisotropy (FA) values ± SD measured in the in the external capsule (EC), internal capsule (IC), corpus callosum (CC) and cortex (Cx) of the EPO, NaCl and Control rats (n = 6 rats for each group, NaCl, EPO and Control, mean values over the four different images planes corresponding to the one of the figure 6; *, ‡, †: *P*<0.05 NaCl vs. Control, EPO vs. Control and EPO vs. NaCl, respectively).

FA measurements were made at four different levels of the brain on axial slice corresponding to the levels of the genu, the body (2 image-planes) and the splenium of the corpus callosum. ROIs are overlaid on direction encoded color maps on figure 6. Results are presented as mean values over the four different levels (Figure 7). In the IC, EC and CC, FA values were found significantly lower in the NaCl group compared with Control group whereas there was no significant difference between EPO and controls. Only in the CC, a significant difference in FA values was observed between EPO and NaCl. In the EC, FA = 0.51±0.02, 0.44±0.05, 0.49±0.03 for Control, NaCl and EPO, respectively; in the IC, FA = 0.49±0.04, 0.41±0.05, 0.45±0.04 for Control, NaCl and EPO, respectively and in the CC, FA = 0.65±0.04, 0.49±0.05, 0.59±0.04 for Control, NaCl and EPO, respectively. This FA decrease was related to a significant increase of the radial diffusivity. In the EC, D_⊥_ = 2.07±0.17×10^−4^ mm^2^.s^−1^, 2.54±0.17×10^−4^ mm^2^.s^−1^, 2.42±0.46×10^−4^ mm^2^.s^−1^ for Control, NaCl and EPO, respectively; in the IC, D_⊥_ = 2.21±0.19×10^−4^ mm^2^.s^−1^, 2.58±0.27×10^−4^ mm^2^.s^−1^, 2.47±0.42×10^−4^ mm^2^.s^−1^ for Control, NaCl and EPO, respectively and in the CC, D_⊥_ = 1.58±0.17×10^−4^ mm^2^.s^−1^, 2.41±0.26×10^−4^ mm^2^.s^−1^, 2.26±0.50×10^−4^ mm^2^.s^−1^ for Control, NaCl and EPO, respectively. In the cortex, no significant difference was found between the groups (NaCl, EPO and Control) neither on diffusivity values, nor on FA (Figure 7).

## Discussion

Using different approaches we aimed at evaluating the protective role of long-term high dose of EPO treatment after moderate HI injury in the immature P3 rat brain. In this study, long-term EPO treatment HI injury in the P3 rat brain did not provide gross cortical grey matter protection but showed partial protective effects on metabolism, white matter structure and somatosensory function. The neurochemical profile of EPO treated rats presented less significant changes than the one of NaCl treated rats. EPO protected the integrity of several white matter tracts as depicted by normalized DTI derived parameters values at P25. From a functional point of view, long-term EPO treatment after P3 HI brain injury promoted whisker stimulated somatosensory functional recovery at P27–P28 to near normal levels.

In a previous study we observed that females rat pups showed smaller injury after HI injury at P3 with better recovery and reduced cortical damage at P25 compared to males [24]. As a consequence of this differential level of damage and recovery, evaluation of EPO effects on HI injury in P3 females would have been difficult to distinguish from the spontaneous recovery in absence of treatment. For this reason, in this therapeutic study we chose to treat and assess male pups only.

There is an important number of published studies of EPO given prior or after a neonatal insult in mice or rats that showed a reduction of the damage with decrease of cell death, not only neurons but also oligodendrocytes with preserved myelination [31], [32]. Furthermore, when administrated acutely after neonatal ischemia, EPO is not only neuroprotective but also stimulates angiogenesis and neurogenesis [39]. It is important to notice that in the majority of these studies, assessment of brain damage was performed a few days after the insult and after short-term treatment only. Moreover, most of these neonatal models of injury did use older pups with a more mature brain at time of injury and treatment. Further, these models are more severe with mild to severe brain damage compared to the one used in this study. Our study investigated a long-term treatment of EPO during 3 weeks with high dose (10 U/g) of EPO during the first week. This treatment regimen does not show similar neuroprotection compared to the shorter therapeutic intervention but it is also difficult to compare as in these studies EPO effect was assessed earlier. A previous study reported that an early administration of EPO over a period of 1 week after HI on P7 rats resulted in reduced infarct volume, significant enhancement of functional revascularization and neuronal replacement in the ischemic hemisphere [15]. In our study, despite early and sustained EPO administration no reduction of infarct size at P25 was seen with any increased neuronal survival measured by NAA concentration and neuronal counts in the EPO group. Moreover, exogenous EPO did not attenuate astrogliosis as depicted by the 50% of EPO treated animals showing significant glial scaring not different from the untreated animals. In the ipsilateral cortices of EPO treated rats, astrogliosis was present and very strong or completely absent. This might be dependent on the extent of the primary injury at P3 as a variability of the lesion size is observed at this time point as depicted by the standard deviation of the percentage of injured cortex at P3. Indeed the degree or nature of the injury itself in the acute phase may elicit differential level of therapeutic effect of EPO. One potential mechanism for these differential actions of EPO under different degrees of injury may be the regulation of EPO-R [40]. In fetal sheep asphyxia model, expression of EPO-R was increased on astrocytes in brief asphyxia insult but required repeated asphyxia insults to be increased on neurons [41]. It also appears that EPO-R expression is different in the core of the injury compared to the penumbra and is mainly increased on apoptotic neurons [42]. Therefore, the moderate HI injury at P3 would lead to a weak increase of EPO receptors on neurons and exogenous EPO would be in excess compared to EPO-R with limited therapeutic effect as not bound to its target.

The presence of astrocytes might hamper the recovery of cortical tissue. High doses of EPO may have an adverse effect as it has been reported that high EPO dosage promotes the proliferation of astrocytes [43]. EPO exerts its neurotrophic role but in the same time activates astrocyte proliferation which accelerates oligodendrocytes maturation that should promote myelin repair [43].

Analyses of MBP staining showed no difference between the EPO and NaCl group neither in the corpus callosum nor in the internal capsule. Again dosage used might be implicated as high-dose of EPO (20 U/g) failed to restore myelination and function after neonatal HI in mice [30]. Nevertheless, in the same regions FA values were restored in the EPO group. The data obtained by *ex-vivo* DTI and SEP recordings showed that EPO has the potential to enhance white matter reorganization and gray matter functional recovery after injury. In the EPO group FA values were restored to values comparable to control in white matter indicating microstructural recovery whereas the NaCl group showed lower FA than control indicating WM tract microstructure disruption. The discrepancy between MBP and microstructural assessment with FA is not surprising as not only the myelin influences FA in white matter but also axonal diameter or axonal compaction [44], [45]. For instance, Favrais *et al.* observed reduced FA in several brain regions following inflammatory injury attributed to axonopathy [46]. Axonal evaluation using electronic microscopy would be necessary to address this question of an effect of EPO on axonal recovery or/and myelin compaction or axonal diameter. Importantly, SEP results indicate that EPO treatment induces functional recovery after HI injury that was not present in the animal that received NaCl only. Indeed, Iwaï *et al.* [47] reported similar results in a neonatal rat model where administration of EPO was ineffective at decreasing infarct volume but significantly improved neurological function outcomes. These findings suggest that gross preservation of neural tissue is not a definite requirement for the recovery of neurological function and that EPO is beneficial for supporting neuronal plasticity and connectivity establishment. The restoration of neurological function is influenced by a variety of factors, such as the reorganization of surviving neuronal tissue, the restoration of myelin and oligodendrocytic architecture and gross anatomical recovery is less a prerequisite than improved connectivity. Chan *et al*. [48] reported 10 weeks after HI injury induced at P9 on mice and without neuroprotective therapy an increased FA in several WM tracts attributed to WM reorganization despite a large loss of grey matter. EPO could stimulate this WM reorganization as depicted by the restoration of FA values in different WM tracts despite no gross anatomical recovery.

## Conclusion

In this study we assessed the effects of long-term high dose of EPO following HI in the P3 pup rat with a multi-modal protocol. Such a protocol is very powerful to evaluate neuroprotective effects of EPO with a wide range of information including functional, macro- and micro-structural, metabolic and histologic assessment. In conclusion, long-term treatment with EPO in the P3 rat brain after HI injury showed functional recovery in the somatosensory cortex, partial white matter microarchitecture preservation and reduction of metabolic alterations despite absence of gross neuroprotection with no cortical volume and neuronal density recovery. This study indicates that further refinements concerning EPO dose and regimen of administration are needed to achieve neuroprotection in the preterm brain.
